# W-MAC: A Workload-Aware MAC Protocol for Heterogeneous Convergecast in Wireless Sensor Networks

**DOI:** 10.3390/s110302505

**Published:** 2011-02-28

**Authors:** Ming Xia, Yabo Dong, Dongming Lu

**Affiliations:** College of Computer Science and Technology, Zhejiang University, No. 38, Zhe-Da Road, Hangzhou, 310027 Zhejiang, China; E-Mails: nmlab_xiaming@zju.edu.cn (M.X.); ldm@zju.edu.cn (D.L.)

**Keywords:** wireless sensor network, heterogeneous convergecast, MAC protocol, TDMA

## Abstract

The power consumption and latency of existing MAC protocols for wireless sensor networks (WSNs) are high in heterogeneous convergecast, where each sensor node generates different amounts of data in one convergecast operation. To solve this problem, we present W-MAC, a workload-aware MAC protocol for heterogeneous convergecast in WSNs. A subtree-based iterative cascading scheduling mechanism and a workload-aware time slice allocation mechanism are proposed to minimize the power consumption of nodes, while offering a low data latency. In addition, an efficient schedule adjustment mechanism is provided for adapting to data traffic variation and network topology change. Analytical and simulation results show that the proposed protocol provides a significant energy saving and latency reduction in heterogeneous convergecast, and can effectively support data aggregation to further improve the performance.

## 1. Introduction

A wireless sensor network (WSN) consists of a large number of low cost, low power sensor nodes that perform data sensing tasks. Convergecast is a typical communication pattern in WSNs, where sensor nodes in the network send data to the sink node periodically. Currently existing MAC protocols for convergecast in WSNs are mostly based on the assumption that each sensor node generates exactly the same amount of data at the same rate. However, in real deployment, this assumption frequently does not hold. Sensor nodes may sense different amounts of data (e.g., they may be equipped with different types or numbers of sensors), or they may have different data reporting frequency configurations. This type of convergecast can be formulated as heterogeneous convergecast, where each sensor node generates different amounts of data in one convergecast operation. In this occasion, existing convergecast MAC protocols can not effectively adapt to variable data traffic on sensor nodes, and cause a great degradation in the overall performance of the network.

This paper presents W-MAC, a workload-aware MAC protocol for heterogeneous convergecast in WSNs. W-MAC employs a subtree-based iterative cascading scheduling mechanism, and a workload-aware time slice allocation mechanism to minimize the power consumption of nodes, while offering a low data latency. In addition, W-MAC provides an efficient schedule adjustment mechanism to adapt to data traffic variation and network topology changes. Analytical and simulation results show that W-MAC outperforms existing protocols in both power consumption and data latency, and can effectively support data aggregation to further improve the performance.

The rest of the paper is organized as follows: Section 2 outlines related work. Section 3 describes our scheduling algorithm and Section 4 provides detailed descriptions on the schedule establishment and adjustment. Section 5 presents the evaluation results and Section 6 concludes the paper.

## 2. Related Work

MAC protocols for WSNs mostly provide wakeup/sleep schedules for sensor nodes to reduce power consumption, and can be roughly categorized as either contention-based [1] or TDMA-based [2]. Although TDMA-based protocols frequently tend to pose a heavier burden on schedule maintenance, they do not suffer from collisions. This is in agreement with recent research showing that TDMA is preferred for the communications in WSNs [3]. There are also other MAC approaches such as CDMA and multi-channel; we will not discuss them in this paper as they typically pose higher requirements on node capability.

In convergecast, if not considering their special data flow direction from sensor nodes to sink nodes, wakeup/sleep schedules will cause the “data forwarding interruption problem” and bring about a high data latency [4]. Therefore, a number of MAC protocols specially designed for convergecast in WSNs have been proposed to alleviate the problem.

DMAC [4] gives the schedule of a node an offset that depends upon its level (the number of hops to the sink node) on the tree. However, DMAC is not collision-free, since nodes in the same level own the same slot to transmit data. To reduce collisions, DMAC requires the node to perform random backoff before trying to transmit data. If the channel is unavailable, the node must wait for 5 slots to retry. MERLIN [5] and QDMAC [6] adopt a similar scheduling rule as in DMAC. As a result, they also suffer from collisions.

In contrast to DMAC, LL-MAC [7] adopts a level-by-level scheduling scheme. It divides the data transfer period into several non-overlapping uniform divisions, and assigns each level of sensor nodes one division. It then allocates each node a set of unique slots from the division to enable collision-free data transfer. Because the data traffic in each level is different, part of the slots are wasted. At the same time, LL-MAC makes the node cache all data records from its children before relaying, thus causes a high memory usage. LL-MAC also considers the scheduling for network control packets (e.g., time synchronization and route discovery) transfer. However, the control interval of LL-MAC is long and requires all nodes to keep awake in the whole control interval, thus consuming excessive energy.

The above protocols do not work well in a heterogeneous convergecast scenario. For DMAC, an extra-large data record must be divided into multiple segments to be transmitted in multiple slots, and the node can only perform transmission once every 5 slots. LL-MAC does not provide extra slots if the data record generated by a node is too long to be transmitted in one slot, as a result, the uniform slot assigned by LL-MAC must be larger than the longest data record and the nodes which have shorter data records cannot fully use their slots. In addition, none of the above protocols can effectively support data aggregation. DMAC will immediately forward the data once received, thus no time is spared for data aggregation. The traffic reduction brought by data aggregation will not benefit the performance of LL-MAC, since its slots allocation is only based on the number of descendants of the node.

There are some other similar works that focus on the data transmission time scheduling in convergecast. They can be divided into two categories: (1) works that aim to minimize the convergecast time or energy cost [8–14]. These methods only realize collision-free transmission in the case that each node generates the same amount of data at the same rate; (2) works that aim to maximize data aggregation efficiency. Some of them [15,16] employ the similar idea as that in DMAC, thus they also suffer from collisions. Some researchers have tried to avoid collisions in data aggregation [17–19], however, these TDMA scheduling approaches restrict the nodes to have to aggregate received packets into one packet to be transmitted in one time slot. As a result, these scheduling approaches are eventually not designed for heterogeneous convergecast, and at the same time, many aggregation methods, especially those lossless aggregation functions such as packing aggregation [20], will be not applicable.

## 3. Scheduling Algorithm for Heterogeneous Convergecast

**Figure 1.**
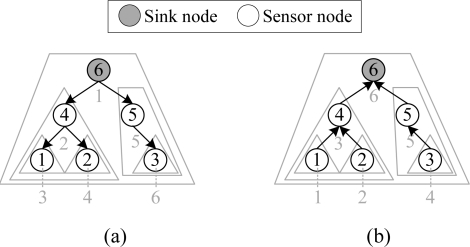
Scheduling sequence in W-MAC: **(a)** control packet delivery and **(b)** data packet delivery.

As mentioned before, level-by-level data transfer as in LL-MAC wastes slots and brings extra data latency. We alternatively took a subtree as the unit, and designed a collision-free iterative cascading scheduling mechanism, which makes nodes perform network control and data delivery operations subtree-by-subtree. The scheduling algorithm is divided into control interval scheduling and data interval scheduling. For the control interval scheduling, the control packet disseminations are performed from the sink node to the furthest sensor nodes (*i.e*., nodes with the largest number of hops) subtree-by-subtree, as in Figure 1(a); for the data interval scheduling, the data packet deliveries are performed from the furthest sensor nodes to the sink node subtree-by-subtree, as in Figure 1(b). Typically, the control packet disseminations are performed first, and then the data packet deliveries will be performed. Another key technique in the scheduling algorithm is the workload-aware time slice allocation mechanism for minimizing the active time of nodes. In our scheduling, the workload of a node is essentially the communication workload of the subtree which is rooted at the node.

The workload *W* of a node is defined as [*WO*, *WR*, *WT*]. *WO* is the time required for the control packet from the node to reach all its descendants, *WR* is the time required for the node to collect data from all its descendants and *WT* is the time required for the node to transmit all data generated by its descendants and itself. *WO* is referred to as control workload; *WR* and *WT* are referred to as data workload.

The scheduling is based on the workloads collected from the children, and the scheduling process only involves the node and its parent. The parent node first allocates a time pool *B* to each child according to the child’s workload *W*. The time pool *B* is defined as [*B^S^*, *B^L^*], in which *B^S^* is the start time of *B*, and *B^L^* is the length of *B*. The time pools are categorized as: control time pool *B_C_*, the time pool for control packet disseminations, and data time pool *B_D_*, the time pool for data packet deliveries. Then the node will calculate and acquire its time slices from the time pool obtained from the parent. The time slices can be categorized as:

♦ *RX*: The time slice for receiving packet. *RX* is defined as [*RX^S^*, *RX^L^*], in which *RX^S^* is the start time of *RX* and *RX^L^* is the length of *RX*. *RX* can be further categorized as *RX* for control interval (*RX_C_*) and *RX* for data interval (*RX_D_*).
♦ *TX*: The time slice for transmitting packet. *TX* is defined as [*TX^S^*, *TX^L^*], in which *TX^S^* is the start time of *TX* and *TX^L^* is the length of *TX*. *TX* can be further categorized as *TX* for control interval (*TX_C_*) and *TX* for data interval (*TX_D_*).

Therefore, the active time of a node in one working cycle can be represented as *RX_C_* ∪ *TX_C_* ∪ {*RX_D_*} ∪ *TX_D_*. One node may have multiple *RX_D_*s, but will have, and only have, one each of the other types of time slices. The node will be kept in sleep in the rest of the time. We will then elaborate on the time pool allocation and time slice calculation algorithms in W-MAC. Other variables used are listed below:

♦ *C_i_*: The set of direct children of the node *u_i_*.
♦ *T_C_*: The time required for transmitting one control packet.

### 3.1. Control Interval Scheduling

The control workload *WO* of a node is determined by the number of descendants of the node. For the node *u_i_*, its control workload *WO_i_* can be calculated from all its children’s control workloads by using Equation (1):

(1) $${\mathrm{WO}}_{i}=\sum_{u_{j}\in C_{i}}{\mathrm{WO}}_{j}+T_{C}$$

After getting all children’s control workloads, the parent can calculate the control time pool allocations for its children. For the node *u_i_*, the control time pool for the child node *u_j_* can be calculated using Equation (2), in which $B_{\mathrm{Ck}}^{L}$ indicates the length of the *k*th node’s control time pool whose allocation is before the node *u_i_*:

(2) $$\{\begin{array}{l} B_{\mathrm{Cj}}^{S}=B_{\mathrm{Ci}}^{S}+T_{C}+\sum_{k=1}^{j-1}B_{\mathrm{Ck}}^{L}\\ B_{\mathrm{Cj}}^{L}={\mathrm{WO}}_{j} \end{array},\forall u_{j}\in C_{i}$$

The node will calculate its *RX_C_* and *TX_C_* locally after obtaining the control time pool allocation from the parent. For the node *u_i_*, ${\mathrm{RX}}_{\mathrm{Ci}}^{S}$ is equal to ${\mathrm{TX}}_{C}^{S}$ of its parent, and ${\mathrm{RX}}_{\mathrm{Ci}}^{L}$ is equal to *T_C_*. ${\mathrm{TX}}_{\mathrm{Ci}}^{S}$ is equal to $B_{\mathrm{Ci}}^{S}$, and ${\mathrm{TX}}_{\mathrm{Ci}}^{L}$ is also equal to *T_C_*.

**Figure 2.**
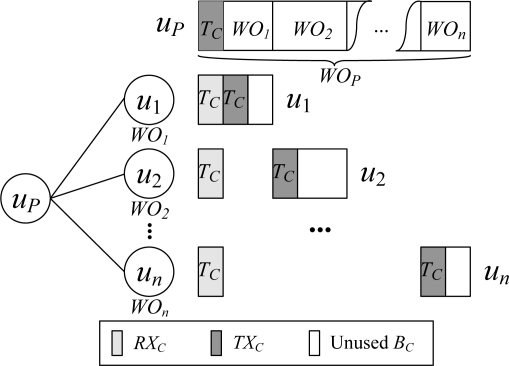
Scheduling for the control interval.

An example of the control interval scheduling process is shown in Figure 2. Let the number of children of the node *u_P_* be *n*, and the control workload of the child node *u_j_* (1 ≤ *j* ≤ *n*) be *WO_j_*. Then *WO_P_* is $\sum_{j=1}^{n}{\mathrm{WO}}_{j}+T_{C}$. In scheduling, *T_C_* amount of time at the beginning of the control time pool of the node *u_P_* is reserved for *u_P_* to transmit control packet, and the rest of the time is allocated to its children. It can be observed that the active time of a node in the control interval is a constant, and achieves the minimum value 2*T_C_*.

### 3.2. Data Interval Scheduling

The data workload *WR* and *WT* of a node is determined by the number of descendants of the node, the amount of data generated by the node and the node’s descendants, and the data aggregation rate on the node. For the node *u_i_*, its data workload *WR_i_* and *WT_i_* can be calculated from all its children’s data workloads by using Equation (3), in which *WTS_i_* represents the time required for transmitting data generated by the node *u_i_* itself, and *R_i_* represents the data aggregation rate on *u_i_*:

(3) $$\begin{array}{l} {\mathrm{WR}}_{i}=\sum_{u_{j}\in C_{i}}\left({\mathrm{WR}}_{j}+{\mathrm{WT}}_{j}\right)\\ {\mathrm{WT}}_{i}=(\sum_{u_{j}\in C_{i}}{\mathrm{WT}}_{j}+{\mathrm{WTS}}_{i})R_{i} \end{array}$$

After getting all children’s data workloads, the parent can calculate the data time pool allocations for its children. For the node *u_i_*, the data time pool for the child node *u_j_* can be calculated using Equation (4), in which $B_{\mathrm{Dk}}^{L}$ indicates the length of the *k*th node’s data time pool whose allocation is before the node *u_i_*:

(4) $$\{\begin{array}{l} B_{\mathrm{Dj}}^{S}=B_{\mathrm{Di}}^{S}+\sum_{k=1}^{j-1}B_{\mathrm{Dk}}^{L}\\ B_{\mathrm{Dj}}^{L}={\mathrm{WR}}_{j}+{\mathrm{WT}}_{j} \end{array},\forall u_{j}\in C_{i}$$

The node will calculate its *RX_D_* and *TX_D_* locally after obtaining the data time pool allocation from the parent. For the node *u_i_*, *RX_Dij_* (the time slice for *u_i_* to receive data from the child node *u_j_*) can be calculated using Equation (5), and *TX_Di_* can be calculated using Equation (6):

(5) $$\{\begin{array}{l} {\mathrm{RX}}_{\mathrm{Dij}}^{S}=B_{\mathrm{Di}}^{S}+\sum_{k=1}^{j-1}B_{\mathrm{Dk}}^{L}+{\mathrm{WR}}_{j}\\ {\mathrm{RX}}_{\mathrm{Dij}}^{L}={\mathrm{WT}}_{j} \end{array},\forall u_{j}\in C_{i}$$

(6) $$\{\begin{array}{l} {\mathrm{TX}}_{\mathrm{Di}}^{S}=B_{\mathrm{Di}}^{S}+{\mathrm{WR}}_{i}\\ {\mathrm{TX}}_{\mathrm{Di}}^{L}={\mathrm{WT}}_{i} \end{array}$$

**Figure 3.**
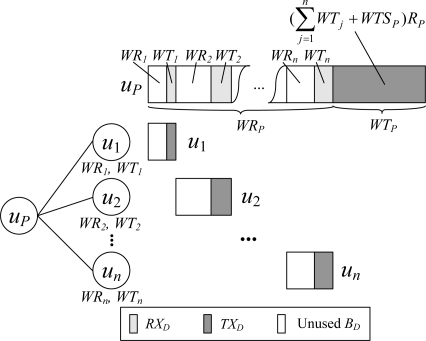
Scheduling for the data interval.

An example of the data interval scheduling process is shown in Figure 3. Let the number of children of the node *u_P_* be *n*, and the data workload of the child node *u_j_* (1 ≤ *j* ≤ *n*) be *WR_j_* and *WT_j_*. Then *WR_P_* is:

$$\sum_{j=1}^{n}({\mathrm{WR}}_{j}+{\mathrm{WT}}_{j}),$$

and *WT_P_* is $(\sum_{j=1}^{n}{\mathrm{WT}}_{j}+{\mathrm{WTS}}_{P})R_{P}$.

In scheduling, *WT_P_* amount of time at the end of the data time pool of the node *u_P_* is reserved for *u_P_* to transmit data packet, and the rest of the time is allocated to its children. It can be observed that the active time of a node in the data interval achieves the minimum value required by the node’s workload, and there is no slots waste problem.

It should be noted that concurrent transmission is not used in our scheduling. The rationale behind this decision is that collision-free concurrent transmission requires the nodes to know the interference relationship between each other, but the overhead of detecting and maintaining the interference relationship is too high in traffic and topology variable heterogeneous convergecast networks. As a result, we chose to let each node own its unique transmission time slice to completely avoid collisions while keeping the scheduling algorithm light-weight.

**Figure 4.**
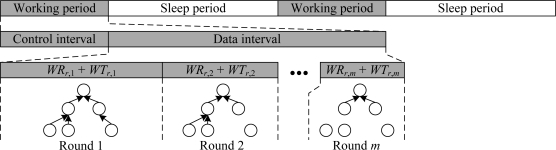
Scheduling of multi-rounds convergecast.

Because the subtree-based iterative cascading scheduling mechanism makes the node transmit data after receiving all data from children, the data aggregation scheme can achieve the highest accuracy and efficiency. However, as in LL-MAC, this attribute may lead to buffer overflow because sensor nodes are typically equipped with limited memory space. In order to alleviate this problem, we can break a single convergecast operation into multiple rounds, and in each round, we transmit only part of the data records. To support multi-rounds convergecast, we extend the data workload of the node *u_i_* to a *m* × 2 matrix ([*WR*_*i*,1_,*WT*_*i*,1_], [*WR*_*i*,2_,*WT*_*i*,2_],…[*WR_i,m_*,*WT_i,m_*]), where *m* indicates the number of rounds. Then, we will have *m* different schedules for one data interval according to the data workload of each round, and the data time pools of the sink node *u_r_* can be represented as:

$$\left[0,{\mathrm{WR}}_{r,1}+{\mathrm{WT}}_{r,1}],[{\mathrm{WR}}_{r,1}+{\mathrm{WT}}_{r,1},{\mathrm{WR}}_{r,2}+{\mathrm{WT}}_{r,2}],...,[\sum_{k=1}^{m-1}{\mathrm{WR}}_{r,k}+{\mathrm{WT}}_{r,k},{\mathrm{WR}}_{r,m}+{\mathrm{WT}}_{r,m}\right]$$

if we take the start time of the data interval as 0, as shown in Figure 4.

## 4. Schedule Establishment and Adjustment

### 4.1. Schedule Establishment

W-MAC makes two assumptions in establishing the schedule: (1) each sensor node knows its parent node; (2) each sensor node knows the amount of data it generates.

Therefore, there are two preliminary actions before establishing the schedule:

♦ Performing route discovery, then the sensor node can get the information about its parent from the routing layer.
♦ Acquiring the amount of data generated by the node itself from the application layer.

These two preliminaries do not have any special requirement to the routing and application layer, and thus our MAC scheduling can be directly applied to existing sensor networks. The schedule establishment can be divided into two steps: workload collection and time pool allocation.

#### 4.1.1. Workload Collection

To initialize the workload collection, the sink node broadcasts a “workload collecting” message in the network. Sensor nodes that receive this message will report workloads to their parents. In order to achieve accurate workload calculation, the children must finish reporting before their parent. Therefore we adopted a mechanism similar to “cascading timeouts” [15] to arrange the workload reporting time of nodes, in which the node with larger hop number will report its workload earlier.

#### 4.1.2. Time Pool Allocation

**Figure 5.**
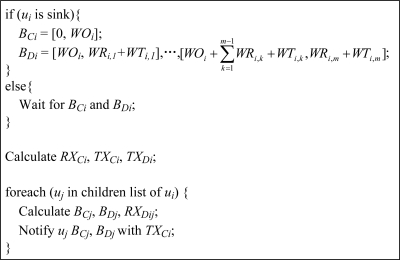
Time pool allocation and time slice calculation.

When the workload collection is finished, the sink node initializes the time pool allocation. Each node calculates its time slices, and allocates time pools to its children according to Figure 5. The overhead of the schedule establishment is quite low because: (1) in the workload collection, each node in the network (including the sink node and sensor node) only stores its children’s workloads, and reports its workload to the parent (if exists); (2) in the time pool allocation, each node only receives the time pool allocation message from its parent (the sink node can generate its time pool), and notifies its children their time pool allocations. All time slices are calculated locally on the nodes. Therefore, the overhead is almost equally distributed to each node in the network.

### 4.2. Schedule Adjustment

Parameters of a WSN system may vary during run time, and this variability will greatly affect the efficiency of the schedule. Variability of the system can be categorized as: (1) data traffic variation. For instance, when the model, number or configuration of the sensors equipped on the node changes, the workload of that node will vary; (2) network topology changes. The unstable nature of wireless communication makes the topology of a WSN prone to frequent changes. Obviously, node insertion or removal will affect the workload of the parent.

If there is no schedule adjustment mechanism, then we have to reestablish the schedule once the workload of a node or a set of nodes changes. Because the energy consumption of schedule reestablishment is relatively high, the protocol will not be able to keep the node working on an energy-efficient manner. We will then proceed to discuss our schedule adjustment mechanism to ensure the efficiency of our protocol in data traffic and network topology variable scenarios.

#### 4.2.1. Data Traffic Variation

The data traffic variation will only affect the data interval scheduling. In W-MAC, when the data traffic of a node varies, the node will stamp its data packet with a “data traffic varies” mark, which contains Δ*WT* (the change of *WT*). Parent node that receives the data packet with this mark will recalculate and record its Δ*WR* (the change of *WR*) and Δ*WT*. Then the parent node will stamp its data packet with a “data traffic varies” mark which contains both Δ*WR* and Δ*WT*.

The process repeats until the data packet with the “data traffic varies” mark reaches the sink. Then the sink will perform schedule adjustment in the next control interval. The sink node will first adjust its time pool allocation according to Equation (7), in which *m* indicates the number of rounds to complete convergecast, and *B_Dr,k_* indicates the data time pool allocation of the sink node *u_r_* for the *k*th round of convergecast:

(7) $$\{\begin{array}{l} {B_{\mathrm{Dr},k}^{S}}^{\prime }=B_{\mathrm{Dr},k}^{S}+\sum_{l=1}^{k-1}\left({\Delta \mathrm{WR}}_{r,l}+{\Delta \mathrm{WT}}_{r,l}\right)\\ {B_{\mathrm{Dr},k}^{L}}^{\prime }=B_{\mathrm{Dr},k}^{L}+{\Delta \mathrm{WR}}_{r,k}+{\Delta \mathrm{WT}}_{r,k} \end{array},\;\;1\leq k\leq m$$

Then, the schedule adjustment operation will be triggered from the sink node to leaf sensor nodes. Let the number of children of the node *u_P_* be *n* (*u_P_*’s children {*u_j_* | 1 ≤ *j* ≤ *n*} are ordered by the sequence in which they appear in the time pool allocation). If the data workload of the child node *u_t_* (1 ≤ *t* ≤ *n*) varies, *u_P_* will adjust the data time pool allocation according to equation (8), and notify the children in sending control packet:

(8) $$\begin{array}{l} \{\begin{array}{l} {B_{\mathrm{Dj},k}^{S}}^{\prime }=B_{\mathrm{Dj},k}^{S}+({B_{\mathrm{Dp},k}^{S}}^{\prime }-B_{\mathrm{Dp},k}^{S})\\ {B_{\mathrm{Dj},k}^{L}}^{\prime }=B_{\mathrm{Dj},k}^{L} \end{array},\;\;\;\mathrm{if}\;\;j<t\\ \{\begin{array}{l} {B_{\mathrm{Dj},k}^{S}}^{\prime }=B_{\mathrm{Dj},k}^{S}+({B_{\mathrm{Dp},k}^{S}}^{\prime }-B_{\mathrm{Dp},k}^{S})\\ {B_{\mathrm{Dj},k}^{L}}^{\prime }=B_{\mathrm{Dj},k}^{L}+{\Delta \mathrm{WR}}_{t,k}+{\Delta \mathrm{WT}}_{t,k} \end{array},\;\;\mathrm{if}\;\;j=t\\ \{\begin{array}{l} {B_{\mathrm{Dj},k}^{S}}^{\prime }=B_{\mathrm{Dj},k}^{S}+({B_{\mathrm{Dp},k}^{S}}^{\prime }-B_{\mathrm{Dp},k}^{S})+{\Delta \mathrm{WR}}_{t,k}+{\Delta \mathrm{WT}}_{t,k}\\ {B_{\mathrm{Dj},k}^{L}}^{\prime }=B_{\mathrm{Dj},k}^{L} \end{array},\;\;\mathrm{if}\;\;j>t \end{array}$$

**Figure 6.**
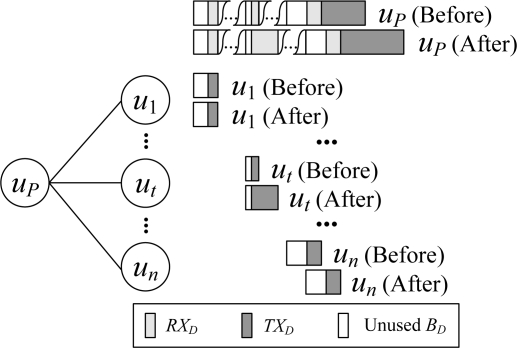
Schedule adjustment for data traffic variation.

The schedule adjustment process of a single round of convergecast is shown in Figure 6.

The node will recalculate its time slices, and further adjust the data time pool allocations of its children when received the data time pool allocation adjustment notification from the parent.

#### 4.2.2. Network Topology Change

The network topology change will affect both the control and data interval scheduling. The network topology changes include: node insertion, node removal and node changing parent.

##### A. Node Insertion

Because the scheduling in W-MAC eliminates the idle listening, it is quite hard to detect the node insertion event. Therefore we appended a very short “child admission” time to the end of *TX_C_*. After receiving a control packet, the new node will return a “node insertion” message, which contains its workload information, to the node that broadcasted the control packet. The node that receives the “node insertion” message will stamp its data packet with a “node insertion” mark, which contains the changes of workloads (Δ*WO*, Δ*WR* and Δ*WT*). The following adjustment process is quite similar to that for data traffic variation, and both the control and data interval scheduling will be adjusted. The new node will keep on listening on the channel after sending the “node insertion” message in this control interval. If a better parent node (mostly determined by routing metrics) is detected, the node changing parent operation will be triggered.

##### B. Node Removal

If a node does not receive any data from one child for several consecutive working cycles, it will stamp its data packet with a “node removal” mark, which contains the changes of workloads (Δ*WO*, Δ*WR* and Δ*WT*). The following adjustment process is similar to that for data traffic variation, and will also adjust both the control and data interval scheduling.

##### C. Node Changing Parent

When a node wishes to change parent, it sends a “node insertion” message in the “child admission” time of the new parent, and proactively sends data packet with the “node removal” mark to the old parent. By this proactive “node removal” notification, we can finish the node changing parent operation within one working cycle as two notification operations are done in one control and data interval, thus the schedule adjustment will not interrupt the data delivery.

The overhead of the proposed schedule adjustment mechanism in W-MAC is very low because: (1) nodes will proactively report workload changes caused by data traffic variation or network topology change, thus no periodical detection is required; (2) when there is a workload change detected, the schedule adjustment process will be triggered, thus schedule reestablishment is never required; (3) most of the adjustment messages are piggybacked on control or data packets, thus the communication overhead brought by the schedule adjustment is minimized.

## 5. Evaluation

In this section, we first present the analysis result of performance of W-MAC, and compare it with LL-MAC to prove that W-MAC can outperform LL-MAC. After that, we test W-MAC on NS2 simulator, and compare it with DMAC and LL-MAC to verify that W-MAC successfully achieves its design goals.

The metrics used in evaluation are listed below:

♦ Power consumption. For DMAC, the energy will only be consumed in data packet transfers; but for LL-MAC and W-MAC, the energy will be consumed in both the data and control packet transfers.
♦ End-to-end latency. The time required for the data from the furthest sensor node to reach the sink node. This term will be referred to as latency unless otherwise stated.
♦ Global latency. For DMAC, this term only includes the length of the data interval; but for LL-MAC and W-MAC, it includes the lengths of both the control and data interval. The global latency determines the maximum data sampling rate supported by the protocol.

### 5.1. Mathematical Analysis

In the discussion below, the data aggregation rate *R* is set to 1 (*i.e*., no data aggregation is employed) since LL-MAC does not support data aggregation. At the same time, we consider the case that each data interval contains only one round of convergecast for simplicity. We denote the number of sensor nodes in the network as *N*, and the data collection cycle (or working cycle) as *T_P_*.

#### 5.1.1. Power Consumption

The power consumption of a sensor node spent on communication (*P*) can be calculated using Equation (9):

(9) $$P=\frac{P_{\mathrm{RX}}T_{\mathrm{RX}}+P_{\mathrm{TX}}T_{\mathrm{TX}}+P_{\mathrm{sleep}}(T_{P}-T_{\mathrm{RX}}-T_{\mathrm{TX}})}{T_{P}}$$

In Equation (9)*P_RX_*, *P_TX_* and *P_sleep_* are the power consumption of a sensor node in receiving data, transmitting data, and sleeping, respectively; *T_RX_* and *T_TX_* are the time spent on receiving and transmitting data. Obviously, we can minimize *P* by minimizing *T_RX_* and *T_TX_*.

For W-MAC, the time spent on receiving data (*T*_*RX*(*W-MAC*)_) and the time spent on transmitting data (*T*_*TX*(*W-MAC*)_) can be calculated using Equation (10), in which *T_A_* is the length of “child admission” time:

(10) $$\begin{array}{l} T_{\mathrm{RX}(W-\mathrm{MAC})}=T_{C}+{2T}_{A}+\sum_{j\in O_{i}}{\mathrm{WTS}}_{j}\\ T_{\mathrm{TX}(W-\mathrm{MAC})}=T_{C}+\sum_{j\in O_{i}}{\mathrm{WTS}}_{j}+{\mathrm{WTS}}_{i} \end{array}$$

For LL-MAC, the time spent on receiving data (*T*_*RX*(*LL-MAC*)_) and the time spent on transmitting data (*T*_*TX*(*LL-MAC*)_) can be calculated using Equation (11):

(11) $$\begin{array}{l} T_{\mathrm{RX}(\mathrm{LL}-\mathrm{MAC})}=(3N+2)T_{C}+K[O_{i}]{\mathrm{WTS}}_{\text{max}}\\ T_{\mathrm{TX}(\mathrm{LL}-\mathrm{MAC})}=T_{C}+(K[O_{i}]+1){\mathrm{WTS}}_{\text{max}} \end{array}$$

In Equation (11), *O_i_* is the set of descendants of the node *u_i_*. *K*[*O_i_*] is the cardinal of *O_i_*. *WTS*_max_ is the length of the uniform slot assigned by LL-MAC, since the length of time slot in LL-MAC depends on the data generation rate of the node that generates the maximum amount of data.

The difference between the power consumption of LL-MAC and W-MAC (Δ*P*) can be calculated using Equation (12), in which *P_LL-MAC_* is the power consumption of LL-MAC, and *P_W-MAC_* is the power consumption of W-MAC. The proof is described in Appendix.

(12) $$\begin{array}{l} \Delta P=P_{\mathrm{LL}-\mathrm{MAC}}-P_{W-\mathrm{MAC}}=\frac{1}{T_{P}}((P_{\mathrm{TX}}-P_{\mathrm{sleep}})(\sum_{j\in O_{i}}\left({\mathrm{WTS}}_{\text{max}}-{\mathrm{WTS}}_{j}\right)+{\mathrm{WTS}}_{\text{max}}-{\mathrm{WTS}}_{i})+\\ \;\;\;\;\;\;\;\;(P_{\mathrm{RX}}-P_{\mathrm{sleep}})((3N+1)T_{C}-{2T}_{A}+\sum_{j\in O_{i}}\left({\mathrm{WTS}}_{\text{max}}-{\mathrm{WTS}}_{j})\right)) \end{array}$$

Because the “node insertion” message is small, *T_A_* is frequently shorter than *T_C_*. If we assume that *T_A_* is equal to *T_C_*, then we have:

(13) $$\begin{array}{ll} \Delta P & =\frac{1}{T_{P}}((P_{\mathrm{TX}}-P_{\mathrm{sleep}})(\sum_{j\in O_{i}}\left({\mathrm{WTS}}_{\text{max}}-{\mathrm{WTS}}_{j}\right)+{\mathrm{WTS}}_{\text{max}}-{\mathrm{WTS}}_{i})+\\ & \left(P_{\mathrm{RX}}-P_{\mathrm{sleep}})((3N-1)T_{C}+\sum_{j\in O_{i}}\left({\mathrm{WTS}}_{\text{max}}-{\mathrm{WTS}}_{j})\right)\right)\\ & \geq \frac{1}{T_{P}}((P_{\mathrm{TX}}-P_{\mathrm{sleep}})(\sum_{j\in O_{i}}\left({\mathrm{WTS}}_{\text{max}}-{\mathrm{WTS}}_{\text{max}}\right)+{\mathrm{WTS}}_{\text{max}}-{\mathrm{WTS}}_{\text{max}})+\\ & \left(P_{\mathrm{RX}}-P_{\mathrm{sleep}})((3N-1)T_{C}+\sum_{j\in O_{i}}\left({\mathrm{WTS}}_{\text{max}}-{\mathrm{WTS}}_{\text{max}})\right)\right)\\ & =(P_{\mathrm{RX}}-P_{\mathrm{sleep}})(3N-1)\frac{T_{C}}{T_{P}} \end{array}$$

Obviously, Δ*P* is always a positive number, *i.e*., W-MAC is more energy efficient than LL-MAC. We can also see that Δ*P* will grow when there is more significant difference between the maximum and average data generation rate of nodes, which shows the supreme energy efficiency of the scheduling of W-MAC in heterogeneous convergecast scenario. At the same time, Δ*P* will also be larger when the number of sensor nodes in the network increases, *i.e*., W-MAC can reserve more energy in large scale network.

#### 5.1.2. Latency

**Figure 7.**
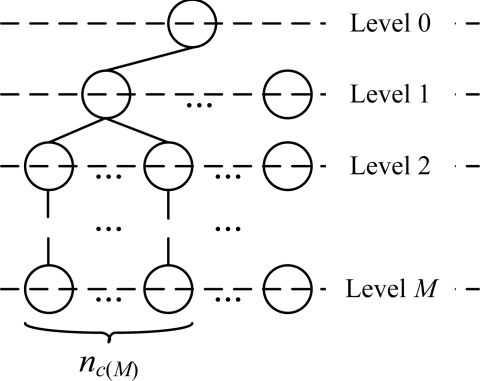
General data collection tree.

Given a general *M*-level data collection network (as shown in Figure 7), we select the node which is at the level *M*, and will be the first one to transmit data in the leftmost subtree, to calculate the end-to-end data transmission latency. We denote the selected node as *u_M0_*, the number of sensor nodes at the level *m* of the subtree which *u_M0_* belongs to as *n*_*c*(*m*)_, and the number of sensor nodes at the level *m* of the whole network as *n*_(*m*)_.

The latency of W-MAC (*D_W-MAC_*) can be calculated using Equation (14):

(14) $$D_{W-\mathrm{MAC}}=(\sum_{i=1}^{M}(i-1)\sum_{j=1}^{n_{c(i)}}{\mathrm{WTS}}_{j})+{\mathrm{WTS}}_{M0}$$

For LL-MAC, its latency (*D_LL-MAC_*) can be calculated using Equation (15) according to its level-by-level scheduling behavior:

(15) $$D_{\mathrm{LL}-\mathrm{MAC}}=((M-1)N+1){\mathrm{WTS}}_{\text{max}}$$

The difference between the latency of LL-MAC and W-MAC (Δ*D*) is:

(16) $$\begin{array}{ll} \Delta D & =D_{\mathrm{LL}-\mathrm{MAC}}-D_{W-\mathrm{MAC}}=\\ & \sum_{i=1}^{M}\left((M-1)n_{\left(i\right)}{\mathrm{WTS}}_{\text{max}}-(i-1)\sum_{j=1}^{n_{c(i)}}{\mathrm{WTS}}_{j}\right)+{\mathrm{WTS}}_{\text{max}}-{\mathrm{WTS}}_{M0}\\ & \geq \sum_{i=1}^{M}(M-i)n_{\left(i\right)}{\mathrm{WTS}}_{\text{max}} \end{array}$$

The proof is described in Appendix. Δ*D* is always a positive number. Similar to the power consumption analysis results, we can see that the latency performance of W-MAC will be much better than that of LL-MAC in large scale heterogeneous convergecast network.

#### 5.1.3. Global Latency

The global latency of W-MAC (*GD_W-MAC_*) can be calculated using Equation (17):

(17) $${\mathrm{GD}}_{W-\mathrm{MAC}}=(N+1)(T_{C}+T_{A})+\sum_{i=1}^{M}i\sum_{j=1}^{n_{\left(i\right)}}{\mathrm{WTS}}_{j}$$

The global latency of LL-MAC (*GD_LL-MAC_*) can be calculated using Equation (18):

(18) $${\mathrm{GD}}_{\mathrm{LL}-\mathrm{MAC}}=(3N+3)T_{C}+{\mathrm{MNWTS}}_{\text{max}}$$

The difference between *GD_LL-MAC_* and *GD_W-MAC_* (Δ*GD*) is:

(19) $$\begin{array}{ll} \Delta \mathrm{GD} & ={\mathrm{GD}}_{\mathrm{LL}-\mathrm{MAC}}-{\mathrm{GD}}_{W-\mathrm{MAC}}=\\ & (2N+2)T_{C}-(N+1)T_{A}+\sum_{i=1}^{M}({\mathrm{Mn}}_{\left(i\right)}{\mathrm{WTS}}_{\text{max}}-i\sum_{j=1}^{n_{\left(i\right)}}{\mathrm{WTS}}_{j}) \end{array}$$

Similarly, if we let *T_A_*=*T_C_*, then Equation (19) can be simplified as:

(20) $$\begin{array}{ll} \Delta \mathrm{GD} & =(N+1)T_{C}+\sum_{i=1}^{M}({\mathrm{Mn}}_{\left(i\right)}{\mathrm{WTS}}_{\text{max}}-i\sum_{j=1}^{n_{\left(i\right)}}{\mathrm{WTS}}_{j})\\ & \geq (N+1)T_{C}+\sum_{i=1}^{M}\left({\mathrm{Mn}}_{\left(i\right)}{\mathrm{WTS}}_{\text{max}}-i\sum_{j=1}^{n_{\left(i\right)}}{\mathrm{WTS}}_{\text{max}}\right)\\ & =(N+1)T_{C}+\sum_{i=1}^{M}(M-i)n_{\left(i\right)}{\mathrm{WTS}}_{\text{max}} \end{array}$$

Δ*GD* is always a positive number. Again, the global latency of W-MAC will be much smaller than that of LL-MAC in large scale heterogeneous convergecast network. We will next examine the performance of W-MAC in the NS2 simulator, and verify the results of the theoretical analysis.

### 5.2. Simulation Results

In our simulation, we use a time-driven data collection network to test the performance of the proposed protocol. In the network, the data collection cycle is set to 1 minute, and the data generation rate of each sensor node is a uniformly distributed random value within the range of 32–512 bytes/minute. Node parameters are set to the typical values of the Crossbow MICAz mote, as shown in Table 1. In LL-MAC and W-MAC, the convergecast in the data interval is broken into 10 rounds to alleviate the buffer usage. We first set the data aggregation rate *R* of W-MAC to 1 in protocols comparison for fair competition, and will later show the performance improvement achieved by combining W-MAC and data aggregation.

#### 5.2.1. Protocol Comparison

**Figure 8.**
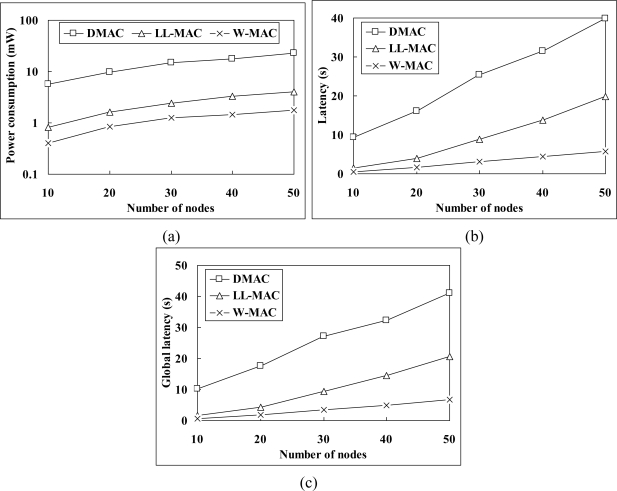
Protocols comparison: **(a)** power consumption; **(b)** latency and **(c)** global latency.

**Figure 9.**
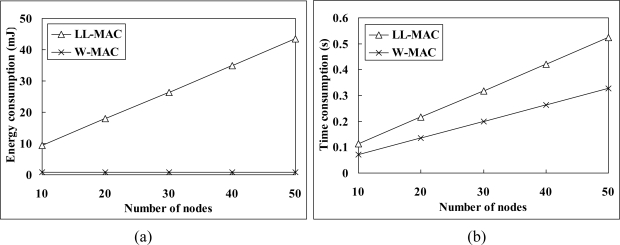
The control overhead of LL-MAC and W-MAC: **(a)** energy consumption and **(b)** time consumption.

Figure 8(a) shows the power consumption simulation results. The average power consumption of W-MAC is only 8% of that of DMAC, and 48% of that of LL-MAC. The excessive power consumption of DMAC mainly comes from the fact that: (1) random backoff increases the length of slot. Meanwhile, the node has to wait for 5 slots before retrying when the channel is unavailable; (2) extra large data record must be divided into multiple segments to be transmitted in multiple slots. The power consumption of LL-MAC is higher than that of W-MAC due to: (1) relatively higher energy consumption in the control interval. Figure 9(a) gives a comparison between the energy consumption of LL-MAC and W-MAC in one control interval. It can be observed that the energy consumption of LL-MAC rises quickly when the scale of the network increases, but the energy consumption of W-MAC is independent of the scale of the network and always keeps at an extremely low level; (2) idle listening caused by uniform slots allocation.

Figure 8(b) shows the simulation results of latency. The average latency of W-MAC is only 11% of that of DMAC, and 33% of that of LL-MAC. The reasons that W-MAC outperforms DMAC in latency are similar to those have been presented in the power consumption simulation results analysis. The latency of LL-MAC is higher than that of W-MAC due to: (1) slots waste problem brought by the level-by-level data transfer scheduling; (2) low channel utilization caused by uniform slots allocation.

Figure 8(c) shows the simulation results of global latency. The average global latency of W-MAC is only 12% of that of DMAC, and 36% of that of LL-MAC. Besides those have been explained in the latency simulation results analysis, the relatively long control interval of LL-MAC also plays an important part in its high global latency. Figure 9(b) gives a comparison between the control interval length of LL-MAC and W-MAC. It can be observed that the control interval length of LL-MAC increases faster when the network scale increases.

**Figure 10.**
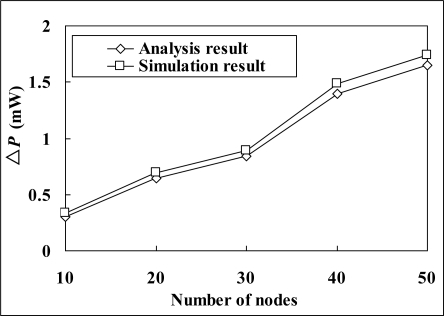
Analysis and simulation result of Δ*P*.

The simulation results verify the correctness of the theoretical analysis. Figure 10 provides a comparison between the analysis and simulation result of Δ*P* (the difference between the power consumption of LL-MAC and W-MAC). We can see that the analysis result matches the simulation result, and there is only a small difference between them. The reason of this small difference is that the node is not always in TX mode in the time slice for transmitting data, but will occasionally switch to RX mode (e.g., to receive acknowledgement). Because the current in TX mode is lower than that in RX mode for the MICAz mote, the analysis result is a little bit smaller than the simulation result. For latency and global latency, the analysis and simulation results completely match with each other.

**Figure 11.**
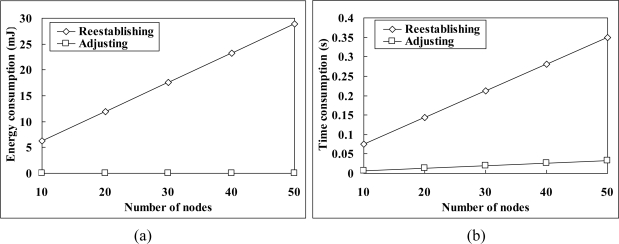
Comparison between the schedule reestablishment and adjustment: **(a)** energy consumption and **(b)** time consumption.

Figure 11 shows the energy and time saving brought by the proposed schedule adjustment mechanism (here we assume that the workload of every node in the network has been changed to make the energy and time consumption of the schedule adjustment mechanism maximized). The period of schedule reestablishment is relatively long, and all nodes have to be kept awake, thus causes extra high energy consumption. As a comparison, through our schedule adjusting, the adjustment messages are piggybacked in normal network control and data delivery packages, and as a result, each node almost does not need to spend additional energy on schedule adjustment, and this advantage can even be kept regardless of the network scale, as shown in Figure 11(a). At the same time, because the schedule adjustment is conducted in normal network control and data delivery operations, the overall time consumption of schedule adjustment is also much lower than that of schedule reestablishment, and increases much slower when the network scale grows, as shown in Figure 11(b).

#### 5.2.2. Collaborating with Data Aggregation

**Figure 12.**
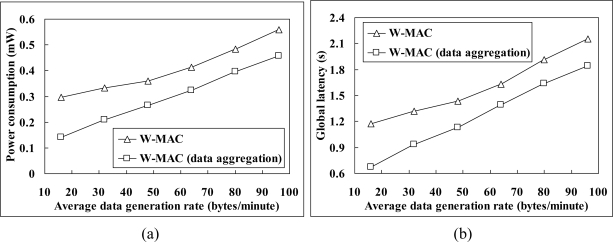
The performance of W-MAC with and without data aggregation: **(a)** power consumption and **(b)** global latency.

Data aggregation is wildly used in WSNs for reducing data traffic. In this part, we choose packing aggregation, a typical lossless data aggregation technique which packs several non-aggregated packets into one aggregated packet without compression, for evaluation. Figure 12 gives a comparison between the performance of W-MAC with and without data aggregation under different data generation rate conditions (The number of nodes is set to 50). When data aggregation is employed, the data aggregation rate *R* in the workload *W* will be reduced, thus W-MAC will shorten the active time of nodes, and then the power consumption and data delivery latency will be lower. The power consumption and global latency of W-MAC with data aggregation can even be reduced to a half of that without data aggregation under low data generation rate condition (16 bytes/minute).

## 6. Conclusions

This paper presents W-MAC, a workload-aware MAC protocol for heterogeneous convergecast in WSNs. W-MAC adopts a subtree-based iterative cascading scheduling mechanism, and a workload-aware time slice allocation mechanism for minimizing the power consumption of nodes while offering a low data latency. We also present a schedule adjustment mechanism for W-MAC to minimize the energy and time consumption in adapting to workload changes, thus ensure the operation efficiency of W-MAC in data traffic and network topology variable scenarios.

Through extensive theoretical analysis and simulation tests, we compared the performance of W-MAC with existing protocols, including DMAC and LL-MAC, and proved that W-MAC successfully meets the design goals. The average power consumption, latency and global latency of W-MAC are only 48%, 33% and 36% of those of the best competitor. Furthermore, W-MAC can effectively support data aggregation to further improve the performance.

## Figures and Tables

**Table 1.** Node Parameters.

**Parameter**	**Value**

RX power	83.1 mW
TX power	66 mW
Sleep power	0.048 mW
Data rate	250 kbps

## Appendix

1. The proof of Equation (12):
  $$\begin{array}{ll} \Delta P & =P_{\mathrm{LL}-\mathrm{MAC}}-P_{W-\mathrm{MAC}}\\ & =\frac{1}{T_{P}}(P_{\mathrm{RX}}((3N+2)T_{C}+K[O_{i}]{\mathrm{WTS}}_{\text{max}})+P_{\mathrm{TX}}(T_{C}+(K[O_{i}]+1){\mathrm{WTS}}_{\text{max}})+\\ & P_{\mathrm{sleep}}(T_{P}-(3N+3)T_{C}-(2K[O_{i}]+1){\mathrm{WTS}}_{\text{max}})-P_{\mathrm{RX}}(T_{C}+{2T}_{A}+\sum_{j\in O_{i}}{\mathrm{WTS}}_{j})-\\ & P_{\mathrm{TX}}(T_{C}+\sum_{j\in O_{i}}{\mathrm{WTS}}_{j}+{\mathrm{WTS}}_{i})-P_{\mathrm{sleep}}(T_{P}-2(T_{C}+T_{A})-2\sum_{j\in O_{i}}{\mathrm{WTS}}_{j}-{\mathrm{WTS}}_{i}))\\ & =\frac{1}{T_{P}}(P_{\mathrm{RX}}((3N+1)T_{C}-{2T}_{A}+\sum_{j\in O_{i}}\left({\mathrm{WTS}}_{\text{max}}-{\mathrm{WTS}}_{j})\right)+P_{\mathrm{TX}}(\sum_{j\in O_{i}}\left({\mathrm{WTS}}_{\text{max}}-{\mathrm{WTS}}_{j}\right)+{\mathrm{WTS}}_{\text{max}}-{\mathrm{WTS}}_{i})+\\ & P_{\mathrm{sleep}}({2T}_{A}-(3N+1)T_{C}-2\sum_{j\in O_{i}}\left({\mathrm{WTS}}_{\text{max}}-{\mathrm{WTS}}_{j})+{\mathrm{WTS}}_{i}-{\mathrm{WTS}}_{\text{max}}\right))\\ & =\frac{1}{T_{P}}(P_{\mathrm{RX}}((3N+1)T_{C}-{2T}_{A}+\sum_{j\in O_{i}}\left({\mathrm{WTS}}_{\text{max}}-{\mathrm{WTS}}_{j})\right)-P_{\mathrm{sleep}}((3N+1)T_{C}-{2T}_{A}+\sum_{j\in O_{i}}\left({\mathrm{WTS}}_{\text{max}}-{\mathrm{WTS}}_{j})\right)+\\ & P_{\mathrm{TX}}(\sum_{j\in O_{i}}\left({\mathrm{WTS}}_{\text{max}}-{\mathrm{WTS}}_{j}\right)+{\mathrm{WTS}}_{\text{max}}-{\mathrm{WTS}}_{i})-P_{\mathrm{sleep}}(\sum_{j\in O_{i}}\left({\mathrm{WTS}}_{\text{max}}-{\mathrm{WTS}}_{j}\right)+{\mathrm{WTS}}_{\text{max}}-{\mathrm{WTS}}_{i}))\\ & =\frac{1}{T_{P}}((P_{\mathrm{TX}}-P_{\mathrm{sleep}})(\sum_{j\in O_{i}}\left({\mathrm{WTS}}_{\text{max}}-{\mathrm{WTS}}_{j}\right)+{\mathrm{WTS}}_{\text{max}}-{\mathrm{WTS}}_{i})+\\ & \left(P_{\mathrm{RX}}-P_{\mathrm{sleep}})((3N+1)T_{C}-{2T}_{A}+\sum_{j\in O_{i}}\left({\mathrm{WTS}}_{\text{max}}-{\mathrm{WTS}}_{j})\right)\right) \end{array}$$
2. The proof of Equation (16):
  $$\begin{array}{ll} \Delta D & =D_{\mathrm{LL}-\mathrm{MAC}}-D_{W-\mathrm{MAC}}=((M-1)N+1){\mathrm{WTS}}_{\text{max}}-(\sum_{i=1}^{M}(i-1)\sum_{j=1}^{n_{c(i)}}{\mathrm{WTS}}_{j})-{\mathrm{WTS}}_{M0}\\ & =\sum_{i=1}^{M}(M-1)n_{\left(i\right)}{\mathrm{WTS}}_{\text{max}}-(\sum_{i=1}^{M}(i-1)\sum_{j=1}^{n_{c(i)}}{\mathrm{WTS}}_{j})+{\mathrm{WTS}}_{\text{max}}-{\mathrm{WTS}}_{M0}\\ & =\sum_{i=1}^{M}\left((M-1)n_{\left(i\right)}{\mathrm{WTS}}_{\text{max}}-(i-1)\sum_{j=1}^{n_{c(i)}}{\mathrm{WTS}}_{j}\right)+{\mathrm{WTS}}_{\text{max}}-{\mathrm{WTS}}_{M0}\\ & \geq \sum_{i=1}^{M}\left((M-1)n_{\left(i\right)}{\mathrm{WTS}}_{\text{max}}-(i-1)\sum_{j=1}^{n_{\left(i\right)}}{\mathrm{WTS}}_{\text{max}}\right)+{\mathrm{WTS}}_{\text{max}}-{\mathrm{WTS}}_{\text{max}}\\ & =\sum_{i=1}^{M}\left((M-1)n_{\left(i\right)}{\mathrm{WTS}}_{\text{max}}-(i-1)n_{\left(i\right)}{\mathrm{WTS}}_{\text{max}}\right)\\ & =\sum_{i=1}^{M}(M-i)n_{\left(i\right)}{\mathrm{WTS}}_{\text{max}} \end{array}$$
